# Are Environmental Factors for Atopic Eczema in ISAAC Phase Three due to Reverse Causation?

**DOI:** 10.1016/j.jid.2018.08.035

**Published:** 2019-05

**Authors:** Charlotte E. Rutter, Richard J. Silverwood, Hywel C. Williams, Philippa Ellwood, Innes Asher, Luis Garcia-Marcos, David P. Strachan, Neil Pearce, Sinéad M. Langan, ISAAC Phase Three Study Group, N. Aït-Khaled, H.R. Anderson, M.I. Asher, R. Beasley, B. Björkstén, B. Brunekreef, J. Crane, P. Ellwood, C. Flohr, S. Foliaki, F. Forastiere, L. García-Marcos, U. Keil, C.K.W. Lai, J. Mallol, E.A. Mitchell, S. Montefort, J. Odhiambo, N. Pearce, C.F. Robertson, A.W. Stewart, D. Strachan, E. von Mutius, S.K. Weiland, G. Weinmayr, H.C. Williams, G. Wong, M.I. Asher, T.O. Clayton, E. Ellwood, P. Ellwood, E.A. Mitchell, A.W. Stewart, C.E. Baena-Cagnani, M. Gómez, M.E. Howitt, J. Weyler, R. Pinto-Vargas, Caja Petrolera de Salud, A.J. D.A. Cunha, L. de Freitas Souza, C. Kuaban, A. Ferguson, D. Rennie, P. Standring, P. Aguilar, L. Amarales, L.A. Benavides, A. Contreras, Y.-Z. Chen, O. Kunii, Q. Li Pan, N.-S. Zhong, G. Aristizábal, A.M. Cepeda, G.A. Ordoñez, C. Bustos, M.-A. Riikjärv, K. Melaku, R. Sa’aga-Banuve, J. Pekkanen, I.E. Hypolite, Z. Novák, G. Zsigmond, S. Awasthi, S. Bhave, N.M. Hanumante, K.C. Jain, M.K. Joshi, S.N. Mantri, A.V. Pherwani, S. Rego, M. Sabir, S. Salvi, G. Setty, S.K. Sharma, V. Singh, T. Sukumaran, P.S. Suresh Babu, C.B. Kartasasmita, P. Konthen, W. Suprihati, M.R. Masjedi, A. Steriu, B.N. Koffi, H. Odajima, J.A. al-Momen, C. Imanalieva, J. Kudzyte, B.S. Quah, K.H. Teh, S. Montefort, M. Baeza-Bacab, M. Barragán-Meijueiro, B.E. Del-Río-Navarro, R. García-Almaráz, S.N. González-Díaz, F.J. Linares-Zapién, J.V. Merida-Palacio, N. Ramírez-Chanona, S. Romero-Tapia, I. Romieu, Z. Bouayad, M.I. Asher, R. MacKay, C. Moyes, P. Pattemore, N. Pearce, B.O. Onadeko, G. Cukier, P. Chiarella, F. Cua-Lim, A. Brêborowicz, D. Solé, M. Sears, V. Aguirre, S. Barba, J. Shah, K. Baratawidjaja, S. Nishima, J. de Bruyne, N. Tuuau-Potoi, C.K. Lai, B.W. Lee, A. El Sony, R. Anderson

**Affiliations:** 1 Faculty of Epidemiology and Population Health, London School of Hygiene and Tropical Medicine, London, UK; 2 Centre of Evidence-Based Dermatology, University of Nottingham, Nottingham, UK; 3 Department of Paediatrics: Child and Youth Health, University of Auckland, Auckland, New Zealand; 4 Pediatric Allergy and Pulmonology Units, Virgen de la Arrixaca University Children’s Hospital, University of Murcia and IMIB Bioresearch Institute, Murcia, Spain; 5 Population Health Research Institute, St George’s University of London, London, UK; 6 Red de Asma, Reacciones Adversas y Alérgicas, Madrid, Spain; 7 Centre for Public Health Research, Massey University, Wellington, New Zealand

**Keywords:** AE, atopic eczema, CI, confidence interval, ISAAC, International Study of Asthma and Allergies in Childhood, OR, odds ratio

## Abstract

Some previously described environmental associations for atopic eczema may be due to reverse causation. We explored the role of reverse causation by comparing individual- and school-level results for multiple atopic eczema risk factors. The International Study of Asthma and Allergies in Childhood (i.e, ISAAC) Phase Three surveyed children in schools (the sampling unit) regarding atopic eczema symptoms and potential risk factors. We assessed the effect of these risk factors on atopic eczema symptoms using mixed-effect logistic regression models, first with individual-level exposure data and second with school-level exposure prevalence. Overall, 546,348 children from 53 countries were included. At ages 6–7 years, the strongest individual-level associations were with current paracetamol use (odds ratio [OR] = 1.45, 95% confidence interval [CI] = 1.37–1.54), which persisted at school-level (OR = 1.55, 95% CI = 1.10–2.21), early-life antibiotics (OR = 1.41, 95% CI = 1.34–1.48), and early-life paracetamol use (OR = 1.28, 95% CI = 1.21–1.36), with the former persisting at the school level, whereas the latter was no longer observed (OR = 1.35, 95% CI = 1.00–1.82 and OR = 0.94, 95% CI = 0.69–1.28, respectively). At ages 13–14 years, the strongest associations at the individual level were with current paracetamol use (OR = 1.57, 95% CI = 1.51–1.63) and open-fire cooking (OR = 1.46, 95% CI = 1.33–1.62); both were stronger at the school level (OR = 2.57, 95% CI = 1.84–3.59 and OR = 2.38, 95% CI = 1.52–3.73, respectively). Association with exposure to heavy traffic (OR = 1.31, 95% CI = 1.27–1.36) also persisted at the school level (OR = 1.40, 95% CI = 1.07–1.82). Most individual- and school-level effects were consistent, tending to exclude reverse causation.

## Introduction

Atopic eczema (AE) prevalence has increased substantially over the last 30 years: up to 20% of children in affluent Western countries have AE during their lives, and prevalence in low-and middle-income countries is increasing (Odhiambo et al., 2009). AE can have a major impact on patients and their families (Balkrishnan et al., 2003, Beattie and Lewis-Jones, 2006).

Although genetic factors clearly play an important role in AE etiology, the dramatic increase in the prevalence of AE in low- and middle-income countries is not consistent with a major role for genetic factors (because these do not change rapidly over time) and strongly suggests that environmental factors are important (Odhiambo et al., 2009, Sandilands et al., 2006).

Phase three of the International Study of Asthma and Allergies in Childhood (ISAAC) has contributed significantly to understanding the associations between single environmental exposures and asthma, AE, and rhinitis (Asher et al., 2010). However, environmental factors may confound each other’s effects in allergic diseases; hence, assessing the role of many key environmental factors together is useful. Findings of cross-sectional studies, including ISAAC, may be limited by reverse causation, whereby the direction of cause and effect is contrary to a common presumption. This arises when having a child at risk of or with AE has led to changes in environmental exposures. For example, parents may remove pets after AE onset if they believe pets exacerbate AE symptoms, resulting in a paradoxical association between increased pet exposure and decreased AE when measured at a single time point (Brunekreef et al., 2012a, Langan et al., 2007), rather than an association between increased pet exposure and increased AE risk. Cross-sectional studies may also be limited by confounding by indication, whereby the association with the risk factor has an alternative explanation; for example, AE may be complicated by skin infections requiring antibiotic treatment, leading to an observed increased association between AE and antibiotic use, rather than antibiotics being on the causal pathway for AE. Confounding by indication has been considered as an alternative explanation in relation to paracetamol (acetaminophen) use and asthma etiology (but not AE) in previous ISAAC reports, although paracetamol may be taken for symptoms of severe skin and other infections associated with AE (Beasley et al., 2008).

In this study, we assessed the effects of the key environmental variables previously each singly associated with AE in ISAAC at an individual level, aiming to find which variables were most important. The individuals in ISAAC were in schools (the sampling unit). Therefore, at the same time, we also incorporated average school-level exposure estimates (calculated from the individual-level data) to assess whether associations seen for these multiple variables at the individual level could be due to bias from reverse causation.

In standard individual-level exposure models, the estimated effect (here an odds ratio [OR]) corresponding to the individual-level risk factor can be interpreted as the OR of the exposed versus the unexposed child, after adjustment for school-level prevalence (as a random intercept). This means that bias due to reverse causation may be a concern where this is plausible, but the estimated effects will not be confounded by unmeasured ecological factors (other environmental factors affecting the whole population).

In school-level exposure prevalence models, the estimated OR corresponding to the school-level prevalence of the risk factor can be interpreted as the effect on an individual of attending a hypothetical school where all children are exposed compared with a hypothetical school where no children are exposed. School-level analyses can suffer from ecological bias, but there is less concern about reverse causation because the actions of a few parents will not significantly affect the school-level prevalence of an exposure. Therefore, comparing the results of these models enables exploration of whether single individual-level risk factors, which could plausibly be due to reverse causation, persist or diminish when explored at the school level.

The complementary approach of individual- and school-level analyses used in this article enables exploration of mutual confounding by environmental factors and different forms of reverse causation, including avoidance bias and confounding by indication.

## Results

### 6–7-year-olds

**Figure 1:**
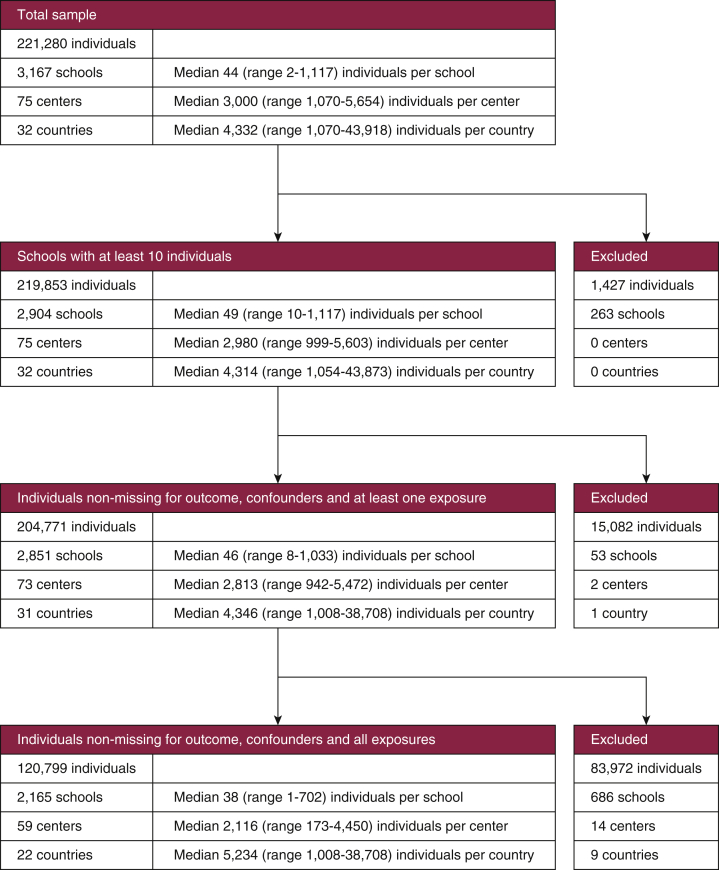
**Atopic eczema data flowchart, ages 6–7 years**. An initial sample of size 221,280 was reduced to 120,799 after exclusions for missing data and small cluster size.

The sample of 6–7-year-olds included 221,280 children (from 3,167 schools, 75 centers, and 32 countries). There were 120,799 children (from 2,165 schools, 59 centers, and 22 countries) with complete data across all analysis variables. See the data flowchart (Figure 1) for further details. Individual- and school-level summary statistics are presented in Table 1 for the common sample and Supplementary Table S1 online for the maximum sample (see “Statistical Analyses” section for definitions).

**Table 1:** Summary Statistics for Participants in the Common Sample

Age Group	Variable	Individual	School Level	
		Prevalence, %	Median Prevalence, %	Prevalence IQR, %
6–7 years Individuals, n = 120,799 Schools, n = 2,165	AE in the last 12 months	7.4	6.4	2.1–12.0
	Farm animals (in utero)	7.7	6.6	2.3–12.7
	Low birth weight	7.7	5.6	2.2–9.7
	Paracetamol (first year)	6.2	70.7	57.1–84.3
	Antibiotics (first year)	5.7	57.1	47.2–65.4
	Breastfed ever	80.5	83.7	73.5–91.8
	Cat (first year)	10.9	8.3	3.6–16.7
	Dog (first year)	19.8	19.7	10.0–30.7
	Farm animals (first year)	9.4	8.3	3.7–14.8
	Two or more siblings	34.7	32.8	18.5–48.4
	Heavy truck traffic (current)	38.0	37.8	27.0–48.7
	Fast food (current)	39.6	31.1	16.5–50.0
	Paternal tobacco (current)	31.8	34.8	21.1–47.8
	Maternal tobacco (current)	16.3	16.7	4.4–32.8
	Paracetamol (current)	18.0	14.2	6.1–25.0
	Open fire cooking (current)	1.9	0.0	0.0–1.6
13–14 years Individuals, n = 233,159 Schools, n = 2,039	AE in the last 12 months	6.2	4.8	2.2–9.1
	Two or more siblings	54.1	59.3	37.7–80.0
	Heavy truck traffic (current)	39.6	39.2	30.1–50.0
	Fast food (current)	53.6	52.8	39.1–68.0
	Paternal tobacco (current)	38.4	37.1	23.8–49.0
	Maternal tobacco (current)	18.3	18.5	3.6–35.4
	Paracetamol (current)	27.0	29.8	17.7–41.7
	Open fire cooking (current)	5.2	0.6	0.0–2.9

The common sample refers to participants with data for atopic eczema symptoms, sex, maternal education, and all exposures of interest.

Abbreviations: AE, atopic eczema; IQR, interquartile range.

Minimally adjusted associations in the common sample were broadly similar to those in the maximum sample (Tables 2, and see Supplementary Table S2 online). The strongest associations in the fully adjusted individual-level analyses were for current paracetamol use (OR = 1.45, 95% confidence interval [CI] = 1.37–1.54), antibiotic use in the first year of life (OR = 1.41, 95% CI = 1.34–1.48), and paracetamol use in the first year of life (OR = 1.28, 95% CI = 1.21–1.36) (Table 2).

**Table 2:** Effects of Individual- and School-Level Exposures on Atopic Eczema Symptoms in the Last 12 Months in the Common Sample

Age Group	Exposure	Individual-Level Exposure		School-Level Exposure	
		Minimally Adjusted^1^ OR (95% CI)	Fully Adjusted^2^ OR (95% CI)	Minimally Adjusted^1^ OR (95% CI)	Fully Adjusted^2^ OR (95% CI)
6–7 years (n = 120,799)	Farm animals (in utero)	1.32 (1.22–1.43)	1.11 (1.00–1.23)	1.48 (1.04–2.12)	1.05 (0.54–2.04)
	Low birthweight	0.92 (0.84–1.01)	0.89 (0.81–0.97)	2.32 (1.43–3.76)	1.78 (1.07–2.95)
	Paracetamol (first year)	1.53 (1.45–1.61)	1.28 (1.21–1.36)	1.11 (0.85–1.46)	0.94 (0.69–1.28)
	Antibiotics (first year)	1.56 (1.49–1.64)	1.41 (1.34–1.48)	1.32 (1.00–1.75)	1.35 (1.00–1.82)
	Breastfed ever	1.09 (1.03–1.16)	1.11 (1.05–1.18)	0.97 (0.69–1.35)	1.06 (0.75–1.48)
	Cat (first year)	1.17 (1.10–1.25)	1.10 (1.03–1.17)	1.40 (0.99–1.97)	1.15 (0.78–1.71)
	Dog (first year)	1.12 (1.07–1.18)	1.05 (1.00–1.11)	1.20 (0.90–1.61)	0.96 (0.69–1.32)
	Farm animals (first year)	1.32 (1.23–1.42)	1.16 (1.06–1.27)	1.50 (1.07–2.10)	1.15 (0.62–2.15)
	Two or more siblings	0.96 (0.91–1.01)	0.95 (0.90–0.99)	1.26 (1.01–1.56)	1.11 (0.88–1.40)
	Heavy truck traffic (current)	1.16 (1.11–1.22)	1.11 (1.06–1.16)	0.92 (0.74–1.14)	0.81 (0.65–1.02)
	Fast food (current)	1.03 (0.98–1.08)	0.99 (0.94–1.04)	0.94 (0.75–1.18)	0.96 (0.76–1.22)
	Paternal tobacco (current)	1.08 (1.03–1.13)	1.04 (0.99–1.10)	1.18 (0.92–1.53)	0.83 (0.61–1.13)
	Maternal tobacco (current)	1.10 (1.04–1.17)	1.06 (0.99–1.13)	1.56 (1.18–2.07)	1.61 (1.14–2.25)
	Paracetamol (current)	1.60 (1.51–1.69)	1.45 (1.37–1.54)	1.63 (1.17–2.26)	1.55 (1.10–2.21)
	Open fire cooking (current)	1.15 (0.97–1.35)	1.12 (0.95–1.32)	2.30 (1.27–4.16)	1.84 (0.98–3.45)
13–14 years (n = 233,159)	Two or more siblings	1.10 (1.05–1.14)	1.08 (1.03–1.12)	1.34 (1.04–1.74)	1.26 (0.97–1.65)
	Heavy truck traffic (current)	1.36 (1.31–1.41)	1.31 (1.27–1.36)	1.66 (1.28–2.17)	1.40 (1.07–1.82)
	Fast food (current)	1.10 (1.05–1.14)	1.05 (1.02–1.10)	2.08 (1.63–2.66)	2.11 (1.66–2.70)
	Paternal tobacco (current)	1.21 (1.16–1.25)	1.15 (1.10–1.19)	0.85 (0.61–1.17)	0.64 (0.44–0.94)
	Maternal tobacco (current)	1.19 (1.14–1.25)	1.11 (1.06–1.16)	0.72 (0.50–1.04)	0.79 (0.52–1.19)
	Paracetamol (current)	1.61 (1.55–1.67)	1.57 (1.51–1.63)	2.68 (1.91–3.75)	2.57 (1.84–3.59)
	Open fire cooking (current)	1.47 (1.33–1.62)	1.46 (1.33–1.62)	2.29 (1.47–3.57)	2.38 (1.52–3.73)

The common sample refers to participants with data for atopic eczema symptoms, sex, maternal education, and all exposures of interest. Mixed logistic regression models with random intercepts at the school, center, and country levels.

Abbreviations: CI, confidence interval; OR, odds ratio.

1 Adjusted for sex and mother’s level of education.

2 Additionally adjusted for all other variables in the table.

In fully adjusted school-level analyses, the associations for current paracetamol use (OR = 1.55, 95% CI = 1.10–2.21) and early-life antibiotic use (OR = 1.35, 95% CI = 1.00–1.82) were maintained, but the association with early-life paracetamol use disappeared (OR = 0.94, 95% CI = 0.69–1.28) (Table 2). Stronger associations were observed at the school level for open-fire cooking (OR = 1.84, 95% CI = 0.98–3.45 vs. OR = 1.12, 95% CI = 0.95–1.32 at the individual level) and maternal tobacco use (OR = 1.61, 95% CI = 1.14–2.25 vs. OR = 1.06, 95% CI = 0.99–1.13 at the individual level). A weak association with current heavy traffic exposure observed at the individual level was not significant at the school level. Associations with breastfeeding were similar in individual- and school-level analyses (OR = 1.11, 95% CI = 1.05–1.18 and OR = 1.06, 95% CI = 0.75–1.48) but with less precision. A potentially harmful association of low birth weight with AE symptoms was seen at the school level (OR = 1.78, 95% CI = 1.07–2.95) compared with a small protective association at the individual level (OR = 0.89, 95% CI = 0.81–0.97) (Table 2).

In analyses stratified by country-level affluence (see Supplementary Tables S3 and S4 online), there was strong evidence at the individual level that being exposed to a cat, dog, or farm animals in the first year of life, or maternal contact with farm animals while pregnant, was associated with AE symptoms in nonaffluent countries at the individual level (see Supplementary Tables S3 and S4), but none of these associations were observed at the school level in either setting. There was also evidence that the association of AE symptoms with current paracetamol was strong at the individual and school levels, with stronger estimates in affluent countries (OR = 1.64, 95% CI = 1.49–1.79) than in nonaffluent settings (OR = 1.35, 95% CI = 1.25–1.45). Weak associations with breastfeeding were observed only at the individual level in affluent countries but were not observed at the school level, with no association seen in nonaffluent countries.

### 13–14-year-olds

**Figure 2:**
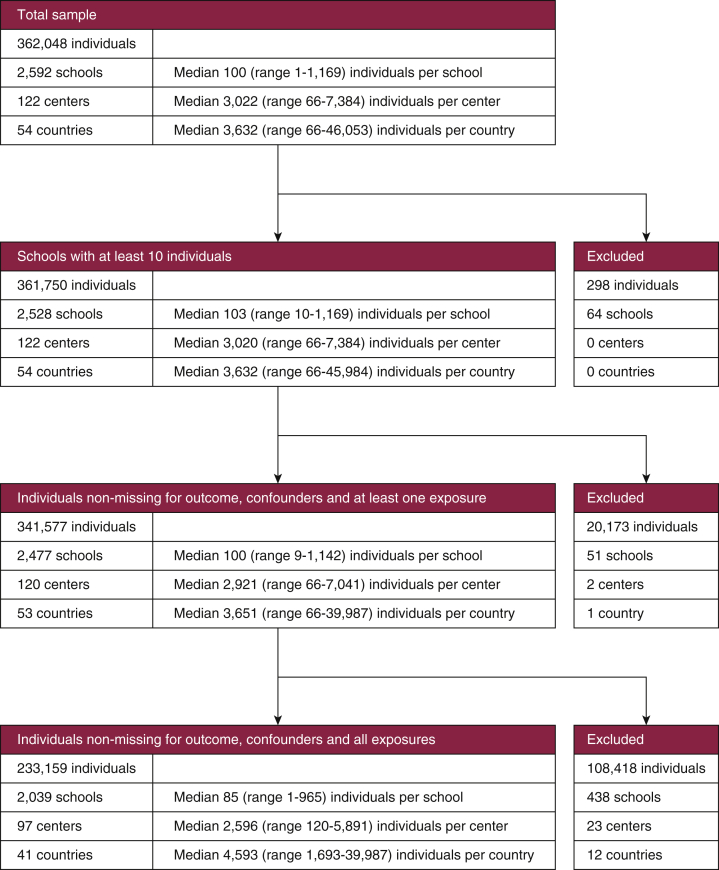
**Atopic eczema data flowchart, ages 13–14 years**. An initial sample of size 362,048 is reduced to 233,159 after exclusions for missing data and small cluster size.

The full 13–14-year-old sample contained 362,048 adolescents (from 2,592 schools, 122 centers, and 54 countries). There were 233,159 adolescents (from 2,039 schools, 97 centers, and 41 countries) with complete data across all analysis variables. See the data flowchart in Figure 2 for further details. Individual- and school-level summary statistics are presented in Table 1 for the common sample and Supplementary Table S1 for the maximum sample.

Minimally adjusted associations in the common sample were broadly similar to those in the maximum sample (Table 2, and see Supplementary Table S3). The strongest associations in fully adjusted individual-level analyses were for current paracetamol use (OR = 1.57, 95% CI = 1.51–1.63), cooking on an open fire (OR = 1.46, 95% CI = 1.33–1.62), and exposure to heavy truck traffic (OR = 1.31, 95% CI = 1.27–1.36) (Table 2).

In fully adjusted school-level analyses, associations for current paracetamol use (OR = 2.57, 95% CI = 1.84–3.59), cooking on an open fire (OR = 2.38, 95% CI = 1.52–3.73) and heavy truck traffic (OR = 1.40, 95% CI = 1.07–1.82) were maintained (Table 2). An association was also observed at the school level for fast food consumption (OR = 2.11, 95% CI = 1.66–2.70), with a much weaker association at the individual level (OR = 1.05, 95% CI = 1.02–1.10). At the individual level there was an association with paternal tobacco use (OR = 1.15, 95% CI = 1.10–1.19), with conflicting findings at the school level (OR = 0.64, 95% CI = 0.44–0.94).

In analyses stratified by country-level affluence (see Supplementary Tables S3 and S4), there was evidence at the individual level that current paracetamol use was slightly more strongly associated with AE symptoms in affluent (OR = 1.75, 95% CI = 1.60–1.92) than nonaffluent (OR = 1.53, 95% CI = 1.47–1.60) countries (see Supplementary Table S3), with stronger associations in both settings at the school level (see Supplementary Table S4). There was also some evidence that paternal tobacco use was associated with AE symptoms in nonaffluent countries (OR = 1.17, 95% CI = 1.12–1.23) at the individual level but not at the school level, and no association was seen in affluent settings (OR = 1.05, 95% CI = 0.96–1.14).

## Discussion

This study is a comprehensive analysis of key risk factors for childhood AE, analyzed together in a multivariable regression analysis, at the individual (child) level to find which variables were most important and at the community (school) level to find which ones remained important; to our knowledge, these findings are previously unreported. The school was the sampling unit, so to our knowledge, analyses using school-level prevalence of exposures offer previously unreported insights into the possible extent of bias due to selective avoidance or confounding by indication. These forms of reverse causation, which are a particular issue in cross-sectional analyses, are less of an issue when using school-level exposures rather than individual-level exposures. When comparing school-level and individual-level findings, if confounding by indication were a major issue, associations would be weaker at the school level, whereas if selective avoidance was the source of reverse causation, associations at the school level would appear to be more harmful. Consistent findings between school- and individual-level analyses suggest that neither of the two forms of reverse causation explain the findings. In contrast, school-level analyses are prone to ecologic (population-level) confounding, which is not an issue when using the individual-level approach. Given that the individual- and school-level analyses will potentially be affected in different ways by reverse causation and confounding by indication, we considered it is sensible to fit regression models at each level (child within school and school within center) and compare the results to assess robustness to different interpretations, rather than considering one approach more appropriate than the other. The analyses use the data from ISAAC Phase Three, in which many individual-level single risk factor analyses found associations, but some of these were not corroborated by our analyses.

### 6–7-year-olds

The results from 6–7-year-olds in this study are summarized and compared with previous ISAAC analyses in Table 3, which also gives an assessment of potential bias and an outline of the biological plausibility of the effect.

**Table 3:** Associations between Eczema Symptoms in the Last 12 months and Risk Factors for the 6–7-Year-Old Age Group Comparing Results from Different Analyses

Exposure	Current Analysis			Previous ISAAC Analysis		Assessment of Bias	Biological Plausibility of Effect
	Individual Level,^1^ OR (95% CI)	School Level,^2^ OR (95% CI)	Comparison	Individual Level,^3^ OR (95% CI)	Comparison with Current Analysis		
Farm animals (in utero)	1.11 (1.00–1.23)	1.05 (0.54–2.04)	No association at school level	1.17 (1.07–1.29) (Brunekreef et al., 2012b)	Consistent	No evidence of reverse causation bias	Not observed at the school level
Low birth weight	0.89 (0.81–0.97)	1.78 (1.07–2.95)	Protective effect at individual level but harmful at school level	0.93 (0.85–1.01) (Mitchell et al., 2014)	Consistent with current individual-level estimate	There could be SES confounding at the community level	Unclear
Paracetamol (first year)	1.28 (1.21–1.36)	0.94 (0.69–1.28)	The significantly harmful effect seen at the individual level does not appear at the school level	1.35 (1.26–1.45) (Beasley et al., 2008)	Consistent with current individual-level estimate	Possible evidence of reverse causation	Unclear
Antibiotics (first year)	1.41 (1.34–1.48)	1.35 (1.00–1.82)	Consistent but weaker	1.42 (1.33–1.51) (Foliaki et al., 2009)	Consistent	Confounding by indication may partly contribute to the association.	Confounding by indication may contribute
Breastfed ever	1.11 (1.05–1.18)	1.06 (0.75–1.48)	Consistent but weaker	1.05 (0.97–1.12) (Bjorksten et al., 2011)	Consistent	No evidence of reverse causation bias	Weak association, biological basis not clear
Cat (first year)	1.10 (1.03–1.17)	1.15 (0.78–1.71)	Consistent	1.09 (1.01–1.17) (Brunekreef et al., 2012a)	Consistent	No evidence of reverse causation bias	—
Dog (first year)	1.05 (1.00–1.11)	0.96 (0.69–1.32)	Consistent	Not available	N/A	No evidence of effect	—
Farm animals (first year)	1.16 (1.06–1.27)	1.15 (0.62–2.15)	Consistent	1.16 (1.07–1.27) (Brunekreef et al., 2012b)	Consistent	No evidence of reverse causation bias	Proposed mechanism related to endotoxin exposure, although unclear
Two or more siblings	0.95 (0.90–0.99)	1.11 (0.88–1.40)	The estimates are in opposing directions, but the individual-level CI is contained in the school-level CI	Categorical^4^ No siblings: 1.00 (ref) One sibling: 1.09 (1.03–1.15) 2 siblings: 1.01 (0.95–1.08) 3+ siblings: 1.04 (0.97–1.12) (Strachan et al., 2015)	Hard to compare because of different models	If there is an effect, it appears small. There is no dose-response relationship (from previous analysis)	—
Heavy truck traffic (current)	1.11 (1.06–1.16)	0.81 (0.65–1.02)	The estimates are in opposing directions, with a harmful effect at the individual level	Categorical^4^ Never: 1.00 (ref) Low: 1.07 (0.99–1.15) Medium: 1.18 (1.09–1.28) Heavy: 1.36 (1.23–1.50) (Brunekreef et al., 2009)	Consistent with the individual level estimate	May relate to bias: parents of children with eczema may move if they are concerned about traffic exposure	Unlikely causal
Fast food (current)	0.99 (0.94–1.04)	0.96 (0.76–1.22)	Consistent	Categorical^4^ Never/occasional: 1.00 (ref) 1–2/week: 1.04 (0.99–1.09) 3+/week: 1.04 (0.95–1.14) (Ellwood et al., 2013)	Consistent	No evidence of effect	—
Paternal tobacco (current)	1.04 (0.99–1.10)	0.83 (0.61–1.13)	The estimates are in opposing directions but the confidence intervals overlap substantially	1.09 (1.04–1.13) (Mitchell et al., 2012)	Consistent with individual-level effect	Very weak association only	No dose-response relationship, unlikely causal
Maternal tobacco (current)	1.06 (0.99–1.13)	1.61 (1.14–2.25)	The school level harmful effect is much greater	1.15 (1.09–1.21) (Mitchell et al., 2012)	Shows a stronger effect than the individual level in the current analysis		No dose-response relationship, unlikely causal
Paracetamol (current)	1.45 (1.37–1.54)	1.55 (1.10–2.21)	Consistent	Categorical^4^ Never/low: 1.00 (ref) Medium: 1.18 (1.08, 1.30) High: 1.87 (1.68, 2.08) (Beasley et al., 2008)	Consistent, the high level is the equivalent to a positive response in the current analysis	No evidence of reverse causation bias	Depletion of glutathione in antigen-presenting cells resulting in a shift from a Th1 to mainly Th2 immune response. (Beasley et al., 2008, Peterson et al., 1998)
Open fire cooking (current)	1.12 (0.95–1.32)	1.84 (0.98–3.45)	Stronger harmful effect seen at school level	1.10 (0.91–1.33) (Wong et al., 2013)	Consistent with individual-level effect from current analysis	Possible avoidance bias, because people with children with AE remove open fires, masking the true magnitude of effect	Persistent AE may be associated with impaired skin barrier and more likely to be affected by aeroallergens and irritants

Abbreviations: AE, atopic eczema; CI, confidence interval; ISAAC, International Study of Asthma and Allergies in Childhood; N/A, not applicable; OR, odds ratio; ref, reference; SES, socioeconomic status; Th, T helper.

1 Fully adjusted for sex, mother’s education level, and all other variables in the table.

2 Fully adjusted for sex, mother’s education level, and school-level prevalence of all other variables in the table.

3 Could be adjusted for a variety of different variables.

4 No direct comparison possible, so closest results are shown.

The strongest associations for 6–7-year-olds in individual-level analyses were for current paracetamol use and antibiotic and paracetamol use in the first year of life. However, in school-level analyses, only associations with current paracetamol persisted. These school-level findings provide evidence against reverse causation, including confounding by indication, as an explanation, and thus make a causal link more likely. Associations between AE and current paracetamol use are consistent with those from individual-level single risk factor analyses in previous ISAAC Phase Three publications, which reported dose-response relationships between the quantity of paracetamol taken in the previous year and current AE symptoms (medium: OR = 1.18, 95% CI = 1.08–1.30 and high: OR = 1.87, 95% CI = 1.68–2.08 compared with no paracetamol) (Beasley et al., 2008). Possible biological mechanisms underlying the observed association between paracetamol use and AE may relate to a depletion of glutathione in antigen presenting cells resulting in a shift from a T helper type 1 to a predominantly T helper type 2 immune response (Beasley et al., 2008, Beasley et al., 2011, Peterson et al., 1998).

Associations with early antibiotic use persisted after adjusting for confounders and were also observed in school-level analyses, although this association was weaker, suggesting that confounding by indication may partly contribute to the association but does not completely explain it. Findings are consistent with those observed in individual-level single risk factor analyses (OR = 1.42, 95% CI = 1.33–1.51) in previous ISAAC Phase Three publications (Foliaki et al., 2009). In our further analyses, stratifying by affluence, similar associations with early antibiotic use were observed in affluent and nonaffluent countries. The reasons for this association with antibiotics and potential causality are unclear, with proposed theories including changes in the gut microbiome (Tsakok et al., 2013).

Breastfeeding was associated with a slightly increased risk of AE at the individual level, with similar but weaker results at the school level. The individual-level association was strongest in affluent countries but was not significant at the school level (see Supplementary Tables S4 and S5). These observations reflect previous reports when assessing breastfeeding as an individual-level exposure in ISAAC Phase Three data (OR = 1.05, 95% CI = 0.97–1.12) (Bjorksten et al., 2011). Our findings do not support the reverse causation theory that in affluent countries, those children at highest risk of developing AE are more likely to be breastfed (Bjorksten et al., 2011, Yang et al., 2009).

We observed evidence of a weak protective effect of low birth weight in individual-level analyses in contrast to a potentially harmful effect in school-level analyses. Individual-level findings are consistent with the previous ISAAC individual-level single risk factor analyses, although additionally these analyses showed no association between birthweight and AE severity, and the importance of the finding from a public health perspective was not clear (Mitchell et al., 2014). It is possible that the opposite school-level association may indicate residual socioeconomic confounding at the community level, because schools with a high proportion of low-birth-weight children may be in more deprived areas (Dibben et al., 2006).

We also observed weak evidence in individual-level analyses that current AE was slightly more common in children exposed to cats, dogs, and farm animals in the first year of life. There were similar results at the school level. In stratified analyses, all of these associations were restricted only to nonaffluent settings, where there is likely to be less awareness of these associations with AE, making bias or differential recall of exposure less likely explanations. Findings for these combined analyses of the ISAAC Phase Three data are consistent with those observed in individual-level single risk factor analyses (Brunekreef et al., 2012a, Brunekreef et al., 2012b).

Current heavy traffic exposure was associated with a weak increased risk of AE symptoms in individual-level but not school-level analyses. A possible explanation for the differential associations at the individual and school levels relates to bias; perhaps parents of children with current AE symptoms are more concerned about heavy traffic exposure and more likely to report it than parents of children without symptoms. These findings may help with interpretation of similar associations observed in previous individual-level single risk factor analyses (Brunekreef et al., 2009).

### 13–14-year-olds

The 13–14-year-olds results from this study are summarized and compared with previous ISAAC analyses in Table 4, along with an assessment of potential bias and an outline of the biological plausibility of the effect.

**Table 4:** Associations between Eczema Symptoms in the Last 12 Months and Risk Factors for the 13–14-Year-Old Age Group, Comparing Results from Different Analyses

Exposure	Current Analysis			Previous ISAAC Analysis		Assessment of Bias	Biological Plausibility of Effect
	Individual Level,^1^ OR (95% CI)	School Level,^2^ OR (95% CI)	Comparison	Individual Level,^3^ OR (95% CI)	Comparison with Current Analysis		
Two or more siblings	1.08 (1.03–1.12)	1.26 (0.97–1.65)	The school level shows a stronger harmful effect, although the CI includes the full individual level CI	Categorical^4^ No siblings: 1.00 (ref) One sibling: 0.91 (0.85–0.98) 2 siblings: 0.96 (0.88–1.03) 3+ siblings: 1.05 (0.97–1.13) (Strachan et al., 2015)	Consistent, although not easy to compare	—	May represent a chance association. No dose-response relationship in individual studies.
Heavy truck traffic (current)	1.31 (1.27–1.36)	1.40 (1.07–1.82)	Consistent	Categorical^4^ Never: 1.00 (ref) Low: 1.08 (0.97–1.19) Medium: 1.30 (1.17–1.45) Heavy: 1.54 (1.37–1.73) (Brunekreef et al., 2009)	Consistent	No evidence of reverse causation bias	Previous studies showed dose-response relationship between levels of exposure to traffic and AE symptoms. No clearly established biological mechanism. The inverse school-level association found in 6–7-year-olds contrasts with the positive school-level association shown here for 13–14-year-olds, suggesting caution should be used when drawing conclusions regarding causality.
Fast food (current)	1.05 (1.02–1.10)	2.11 (1.66–2.70)	The school level shows a stronger harmful effect	Categorical^4^ Never/occasional: 1.00 (ref) 1–2/week: 1.04 (0.99–1.10) 3+/week: 1.20 (1.11–1.28) (Ellwood et al., 2013)	Consistent with individual-level effect in current analysis	Possible avoidance bias, because people with adolescents with AE avoid fast food, masking the true magnitude of effect	Not fully understood, theories around ingested fatty acids and inflammation
Paternal tobacco (current)	1.15 (1.10–1.19)	0.64 (0.44–0.94)	The estimates are in opposing directions, but the confidence intervals overlap substantially; school-level estimates look protective.	1.19 (1.14–1.25) (Mitchell et al., 2012)	Consistent with individual-level effect in current analysis	The finding might support differential reporting of tobacco exposure in those with current AE symptoms or ecologic bias at the school level.	—
Maternal tobacco (current)	1.11 (1.06–1.16)	0.79 (0.52–1.19)	The estimates are in opposing directions, but the confidence intervals overlap substantially	1.22 (1.16–1.28) (Mitchell et al., 2012)	Stronger effect than current individual-level analysis	As for paternal tobacco	—
Paracetamol (current)	1.57 (1.51–1.63)	2.57 (1.84–3.59)	The school-level harmful effect is much greater	Categorical^4^ Never/low: 1.00 (ref) Medium: 1.31 (1.21–1.42) High: 1.99 (1.82–2.16) (Beasley et al., 2011)	Consistent with individual-level current analysis (high is the same as the positive value in current analysis)	Some evidence of possible avoidance bias, masking the true magnitude of the harmful effect	Possible biological mechanisms underlying the observed association between paracetamol use and AE may relate to a depletion of glutathione in antigen-presenting cells, resulting in a shift from a Th1 to a predominantly Th2 immune response (Beasley et al., 2011, Peterson et al., 1998).
Open fire cooking (current)	1.46 (1.33–1.62)	2.38 (1.52–3.73)	Stronger harmful effect seen at school level	1.37 (1.13–1.66) (Wong et al., 2013)	Consistent with individual-level effect in current analysis	Possible avoidance bias, because people with asthmatic children remove open fires, masking the true magnitude of effect	Persistent AE may be associated with impaired skin barrier and more likely to be affected by aeroallergens and irritants.

Abbreviations: AE, atopic eczema; CI, confidence interval; ISAAC, International Study of Asthma and Allergies in Childhood; OR, odds ratio; ref, reference; Th, T helper.

1 Fully adjusted for sex, mother’s education level, and all other variables in the table.

2 Fully adjusted for sex, mother’s education level, and school-level prevalence of all other variables in the table.

3 Could be adjusted for a variety of different variables.

4 No direct comparison possible, so closest results are shown.

The strongest association with current AE in adolescents at the individual level was with current paracetamol use, with even stronger potentially harmful associations observed at the school level. The stronger school-level associations suggest that reverse causation is unlikely to explain these associations, although ecological confounding, whereby confounding arises because of within-area heterogeneity of exposures, is possible. Findings are consistent with previous individual-level single risk factors analyses (Beasley et al., 2011).

Using open fires for cooking was more strongly associated with current AE symptoms at the school level than at the individual level, findings that could be partially attributed to avoidance behavior in parents of children with current AE (Wong et al., 2013). The association with AE observed at the individual level at ages 13–14 years with no association at ages 6–7 years is consistent with previous single exposure ISAAC studies (Wong et al., 2013).

Strong potentially harmful associations with AE symptoms were seen for current heavy traffic exposure at the individual and school levels. This is in contrast to the younger age-group; a possible explanation is that persistent AE may be more severe and more likely to be affected by aeroallergens and irritants. Individual-level analyses showed similar associations with a dose-response relationship between levels of exposure to traffic and AE symptoms (Brunekreef et al., 2009).

Although weak associations were observed at the individual level with current maternal and paternal tobacco exposure, at the school level, the effect was reversed with weak evidence of a protective effect for paternal smoking. This finding might support differential reporting of tobacco exposure in those with current AE symptoms or ecological bias at the school level (Mitchell et al., 2012).

Strong associations were observed at the school level with fast food consumption, with very weak associations being observed at the individual level. Findings are consistent with previous individual-level single risk factor analyses and might plausibly be important for the etiology of AE, although ecologic bias and residual confounding are alternative possibilities (Ellwood et al., 2001, Ellwood et al., 2013).

Weak associations were observed between having two or more siblings and current AE symptoms at an individual level, with slightly stronger associations at the school level (but with weaker precision). Findings are not consistent with those observed at ages 6–7 years, are in contrast to protective associations reported in individual-level single risk factor analyses, and may represent a chance association (Strachan et al., 2015).

### Strengths and limitations

The ISAAC study had worldwide coverage and a very large sample size, including countries from less affluent settings, thus facilitating the study of environmental factors in varied settings (Odhiambo et al., 2009). The use of standardized and validated methods of symptom reporting is a particular strength of the ISAAC study (Flohr et al., 2009). Although self-reported symptoms may be prone to misclassification, they avoid major diagnostic differences because of access to care in different countries and settings, where relying on doctor diagnosis may be more problematic. Selection bias is an unlikely explanation for the findings, because response rates of the children were high (85%).

Assessment of exposures was based on parental or guardian (6–7-year-old children) and study participant (13–14-year-old adolescents) completion of questionnaires about historical exposures rather than objective measures, leading to possible misclassification, which for different exposures may be nondifferential or may be prone to recall biases or reverse causation. Schools were the sampling unit, with individual children of the age group responding within the school, and this structure of the cross-sectional survey enabled these analyses.

Both individual-level and school-level analyses may be biased by residual confounding by factors that were either imperfectly measured or not measured at all; however, because the unmeasured confounders are likely to be different at the school and individual levels, the consistency of findings at both levels is reassuring against associations being due to residual confounding.

## Conclusions

We have further enhanced the ISAAC analyses by using school-level and individual-level exposures, thus allowing us to explore whether specific findings may be due to reverse causation, including confounding by indication. Despite plausible mechanisms, we did not observe findings supportive of selective avoidance in relation to furry pet exposure. The consistent associations between current paracetamol exposure in both age groups and at both the individual and school levels argues against reverse causation as the sole explanation. If paracetamol use in early childhood does have a direct biological role in the development of AE and related disorders such as asthma, then reducing paracetamol use in infancy could reduce the incidence of such diseases. Indeed, a randomized controlled prevention trial in New Zealand called PIPPA Tamariki (ACTRN12618000303246), which seeks to determine whether ibuprofen instead of paracetamol for fever/pain in infancy reduces the incidence of asthma and eczema, is already underway.

Some individual-level single risk factor associations previously identified in ISAAC Phase Three data were not corroborated in the present analyses, but several were: current paracetamol use at ages 6–7 and 13–14 years, early life antibiotic exposure and AE at ages 6–7 years, and current heavy road traffic and open fire cooking and AE symptoms at 13–14 years. The approach of using school-level exposure estimates provides insight that some of the previously reported associations in ISAAC Phase Three studies may be due to reverse causation, but that paracetamol use is unlikely to be explained in this way.

## Material and Methods

### Study

A detailed description of the ISAAC Phase Three methods can be found elsewhere (Ellwood et al., 2005), and they will be briefly summarized here. ISAAC Phase Three is a multicenter, multicountry, cross-sectional study of two age groups of schoolchildren (6–7-year-old children and 13–14-year-old adolescents) chosen from a random sample of schools in a defined geographical area (Asher et al., 1995). The Phase Three survey included a standardized symptom questionnaire, which obtained data on symptoms of asthma, rhinoconjunctivitis, and AE (Asher et al., 1995). It also included a supplementary questionnaire that obtained data on a wide range of possible risk factors for the development of allergic disorders (Beasley et al., 2008). Parents or guardians completed the questionnaires for 6–7-year-olds, and 13–14-year-olds answered the questionnaires themselves. Center eligibility is described in the Supplementary Materials online.

### Variables

The outcome of interest, AE symptoms in the last 12 months, was defined by positive responses to the questions *Has your child/have you ever had an itchy rash which was coming and going for at least six months?*, *Has your child/have you had this itchy rash at any time in the last 12 months?*, and *Has this itchy rash at any time affected any of the following places: the folds of the elbows, behind the knees, in front of the ankles, under the buttocks, or around the neck, ears or eyes?*

Analyses in this article included only the key environmental variables previously each singly associated with AE in ISAAC at an individual level. Full definitions of the environmental risk factors are in Supplementary Table S5 online. Additionally, the analysis considered confounding by sex and highest level of maternal education (primary, secondary, tertiary, or missing/not stated). Finally, stratification by affluence of country was achieved using standard approaches (see the Supplementary Materials) and information on GNI (World Bank, 2016a; World Bank, 2016b; Central Intelligence Agency, 2002).

### Statistical analyses

The two age groups were analyzed separately. All analyses were conducted using mixed-effect logistic regression models. There are four hierarchies of data in the study design: individual, school, center, and country. We accounted for this by including random intercepts at each of the higher three levels. Sex and highest level of maternal education were adjusted for as individual-level confounders in all models. The school-level prevalence of each risk factor was calculated as the proportion of children with that risk factor out of all children included in the analysis in that school.

Separate models were used to assess the effects of individual-level exposures and aggregated school-level prevalence of exposures on the individual-level outcome. Using the approach proposed by Begg and Parides (2003), these effects were formally compared within a multilevel framework, by fitting hybrid fixed-effect models. Results from these models were consistent with a simpler approach and are not discussed further.

In each of these approaches, a minimally adjusted model was fitted. This was done on two samples: (i) the maximum sample, which was the subsample that had no data missing for AE, the confounders (sex, level of maternal education), and the one exposure of interest, and (ii) the common sample, which was the subsample that had no data missing for AE, confounders, and all exposures of interest. A fully adjusted model was also fitted to the common sample. Fully adjusted models included all risk factors at the individual level for the individual-level models and school-level prevalence of all the risk factors for the school-level models.

The extent of co-linearity in fully adjusted models was examined by comparing the standard errors in the fully adjusted model with the standard errors in the minimally adjusted model (common sample). Fully adjusted analyses were additionally stratified by affluent and nonaffluent countries to assess whether avoidance behavior may have contributed to observed associations (because such behavior is more likely in more affluent countries). Effect modification by country-level affluence was tested for each risk factor separately.

All analyses were conducted using Stata, version 14.2 (StataCorp, College Station, TX). Informed consent was obtained from parents of all participating children; the ISAAC Phase Three study was approved by local institutional review boards in all participating centers. Centers obtained their own ethics approval, and it was up to the individual ethics committees to articulate to the investigator which consent method they required. For the 13–14-year-old age group, some centers obtained written consent, and some centers obtained passive consent. Passive consent was permitted when the questionnaires were anonymous and there was no identifying information on the questionnaire. Parents were sent a letter with the questionnaire for them to fill in and return. The letter explained that each 13–14-year-old would complete the survey in class and if they or their son/daughter did not wish to participate, they could phone the investigator to inform them. For the 6–7-year-olds, parents were sent a letter with the questionnaires. If these were completed and returned, consent was implicit.

## ORCIDs

Charlotte Rutter: http://orcid.org/0000-0002-9823-7932

Richard J Silverwood: http://orcid.org/0000-0002-2744-1194

Hywel C Williams: http://orcid.org/0000-0002-5646-3093

Philippa Ellwood: http://orcid.org/0000-0002-1994-4023

Innes Asher: http://orcid.org/0000-0003-1768-3832

Luis Garcia-Marcos: http://orcid.org/0000-0002-0925-3851

David P Strachan: http://orcid.org/0000-0001-7854-1366

Neil Pearce: http://orcid.org/0000-0002-9938-7852

Sinéad M Langan: http://orcid.org/0000-0002-7022-7441

## Conflict of Interest

The authors declare no conflict of interest.

## ISAAC Phase Three Study Group

### ISAAC Steering Committee

N Aït-Khaled∗ (International Union Against Tuberculosis and Lung Diseases, Paris, France); HR Anderson (Population Health Research Institute, St George’s, University of London, UK); MI Asher (Department of Paediatrics: Child and Youth Health, Faculty of Medical and Health Sciences, University of Auckland, New Zealand); R Beasley∗ (Medical Research Institute of New Zealand, Wellington, New Zealand); B Björkstén∗ (Institute of Environmental Medicine, Karolinska Institutet, Stockholm, Sweden); B Brunekreef (Institute of Risk Assessment Science, Universiteit Utrecht, Netherlands); J Crane (Wellington Asthma Research Group, Wellington School of Medicine, New Zealand); P Ellwood (Department of Paediatrics: Child and Youth Health, Faculty of Medical and Health Sciences, University of Auckland, New Zealand); C Flohr (Unit for Population-Based Dermatology Research, St John's Institute of Dermatology, Guy's and St Thomas’ NHS Foundation Trust and King's College London, London, UK); S Foliaki∗ (Centre for Public Health Research, Massey University, Wellington, New Zealand); F Forastiere (Department of Epidemiology, Local Health Authority, Rome, Italy); L García-Marcos (Pediatric Allergy and Pulmonology Units, Virgen de la Arrixaca University Children’s Hospital, University of Murcia and Bio-health Research Institute of Murcia (IMIB), Murcia, Spain); U Keil∗ (Institut für Epidemiologie und Sozialmedizin, Universität Münster, Germany); CKW Lai∗ (Department of Medicine and Therapeutics, The Chinese University of Hong Kong, SAR China); J Mallol∗ (Department of Respiratory Medicine, University of Santiago de Chile, Chile); EA Mitchell (Department of Paediatrics: Child and Youth Health, Faculty of Medical and Health Sciences, University of Auckland, New Zealand); S Montefort∗ (Department of Medicine, University of Malta, Malta), J Odhiambo†∗ (Centre Respiratory Diseases Research Unit, Kenya Medical Research Institute, Nairobi, Kenya); N Pearce (Faculty of Epidemiology and Population Health, London School of Hygiene and Tropical Medicine, London, UK); CF Robertson (Murdoch Children’s Research Institute, Melbourne, Australia); AW Stewart (Population Health, Faculty of Medical and Health Sciences, University of Auckland, New Zealand); D Strachan (Population Health Research Institute, St George’s, University of London, UK); E von Mutius (Dr von Haunerschen Kinderklinik de Universität München, Germany); SK Weiland† (Institute of Epidemiology, University of Ulm, Germany); G Weinmayr (Institute of Epidemiology and Medical Biometry, University of Ulm, Germany); HC Williams (Centre of Evidence-Based Dermatology, University of Nottingham, UK); G Wong (Department of Paediatrics, Prince of Wales Hospital, Hong Kong, SAR China).

### ISAAC International Data Centre

MI Asher, TO Clayton†, E Ellwood, P Ellwood, EA Mitchell, Department of Paediatrics: Child and Youth Health, and AW Stewart, School of Population Health, Faculty of Medical and Health Sciences, The University of Auckland, New Zealand.

### ISAAC Principal Investigators

Argentina: CE Baena-Cagnani∗†, Catholic University of Córdoba (Córdoba), M Gómez, Ayre Foundation; Hospital San Bernardo (Salta); Barbados: ME Howitt∗, Carlton Clinic (Barbados); Belgium: J Weyler, University of Antwerp (Antwerp); Bolivia: R Pinto-Vargas∗, Caja Petrolera de Salud (Santa Cruz); Brasil: AJ da Cunha, Federal Universtity of Rio de Janeiro (Nova Iguaçu), L de Freitas Souza, Universidade Federal da Bahia (Feira de Santana, Salvador, Vitória da Conquista); Cameroon: C Kuaban∗, University of Yaounde (Yaounde); Canada: A Ferguson, University of British Columbia (Vancouver), D Rennie, University of Saskatchewan (Saskatoon); Channel Islands: P Standring, Princess Elizabeth Hospital (Guernsey); Chile: P Aguilar, Hospital CRS El Pino (South Santiago), L Amarales, Regional Hospital “Lautaro Navarro” (Punta Arenas), LA Benavides, (Calama), A Contreras, Hospital de Castro (Chiloe); China: Y-Z Chen∗, Training Hospital for Peking University (Beijing, Tong Zhou), O Kunii, University of Tokyo (Tibet), Q Li Pan, Xinjiang Children's Hospital (Wulumuqi), NS Zhong, Guangzhou Institute of Respiratory Disease (Guangzhou); Colombia: G Aristizábal, Instituto de Enfermedades Respiratorias del Niño S.A. (Bogotá), AM Cepeda, Universidad Metropolitana (Barranquilla), GA Ordoñez, Universidad Libre de Cali (Cali); Ecuador: C Bustos, Hospital Alcivar (Guayaquil); Estonia: M-A Riikjärv∗, Tallinn Children's Hospital (Tallinn); Ethiopia: K Melaku, Addis Ababa University (Addis Ababa); Fiji: R Sa'aga-Banuve, UNICEF (Suva); Finland: J Pekkanen∗, National Public Health Institute (Kuopio County); Gabon: IE Hypolite∗, (Port-Gentil); Hungary: Z Novák, University of Szeged (Szeged), G Zsigmond∗, Senior Consultant (Svábhegy); India: S Awasthi, King George's Medical University (Lucknow), S Bhave, KEM Hospital Research Centre (Rasta Peth), NM Hanumante, Ruby Hall Clinic (Pune), KC Jain, Pioneer Medical Centre (Jodhpur), MK Joshi, Panjat Hospital (Mumbai (16)), VA Khatav, Dr Khatav's Mother and Child Hospital (Borivali), SN Mantri, Jaslok Hospital & Research Centre (Mumbai (29)), AV Pherwani, P.D. Hinduja Hospital and Medical Research Centre (Mumbai (18)), S Rego, St John`s Medical College & Hospital (Bangalore), M Sabir, Maharaja Agrasen Medical College Agroha (Bikaner), S Salvi, Chest Research Foundation (Nagpur, Pimpri), G Setty, (Chennai), SK Sharma, All India Institute of Medical Sciences (New Delhi (7)), V Singh, Asthma Bhawan (Jaipur), T Sukumaran, Pushpagiri Institute of Medical Sciences and Research Center [PIMS], Thiruvalla (Kottayam), PS Suresh Babu, Bapuji Child Health Institute and Research Centre (Davangere); Indonesia: CB Kartasasmita, Padjajaran University (Bandung), P Konthen†, Airlangga University (Bali), W Suprihati, Diponegoro University (Semarang); Iran: MR Masjedi∗, National Research Institute of Tuberculosis and Lung Diseases (Rasht,Tehran); Isle Of Man: A Steriu, Freelance Public Health Consultant, Carol Davila Medical School, Bucharest (Isle of Man); Ivory Coast: BN Koffi∗, (Urban Cote d Ivoire); Japan: H Odajima, National Hospital Organization Fukuoka Hospital (Fukuoka); Kuwait: JA al-Momen, Al-Amiri Hospital (Kuwait); Kyrgyzstan: C Imanalieva∗, Kyrgyz Scientific Research Institute of Obstetrics and Pediatrics (Balykchi, Bishkek); Lithuania: J Kudzyte∗, Kaunas Medical University (Kaunas); Malaysia: BS Quah, Melaka-Manipal Medical College, (Kota Bharu), KH Teh, Hospital Alor Setar (Alor Setar); Malta: S Montefort∗, University of Malta (Malta); Mexico: M Baeza-Bacab∗, University Autónoma de Yucatán (Mérida), M Barragán-Meijueiro, Colegio Mexicano de Alergia, Asma e Inmunología Pediátrica [CoMAAIPE] (Ciudad de México (3)), BE Del-Río-Navarro, Hospital Infantil de México (Ciudad de México (1)), R García-Almaráz, Hospital Infantil de Tamaulipas (Ciudad Victoria), SN González-Díaz, Hospital Universitario (Monterrey), FJ Linares-Zapién, Centro De Enfermedades Alergicas Y Asma de Toluca (Toluca), JV Merida-Palacio, Centro de Investigacion de Enfermedades Alergicas y Respiratorias (Mexicali Valley), N Ramírez-Chanona, Colegio Mexicano de Pediatras Especialistas en Inmunología Clínica y Alergia [COMPEDIA] (Ciudad de México (4)), S Romero-Tapia, Hospital de Alta Especialidad del Niño (Villahermosa), I Romieu, International Agency for Research on Cancer (Cuernavaca); Morocco: Z Bouayad∗, Service des Maladies Respiratoires (Boulmene, Casablanca, Marrakech); New Zealand: MI Asher∗, University of Auckland (Auckland), R MacKay, Canterbury Health Laboratories (Nelson), C Moyes, Whakatane Hospital (Bay of Plenty), P Pattemore, University of Otago, Christchurch (Christchurch), N Pearce, London School of Hygiene and Tropical Medicine (Wellington); Nigeria: BO Onadeko, (Ibadan); Panamá: G Cukier∗, Hospital Materno Infantil Jose Domingo de Obaldia (David-Panamá); Peru: P Chiarella∗, Universidad Peruana de Ciencias Aplicadas [UPC] (Lima); Philippines: F Cua-Lim∗†, University of Santo Tomas (Metro Manila); Poland: A Brêborowicz, Univers.

### Additional ISAAC National Coordinators

Brazil: D Solé, Universidade Federal de São Paulo; Canada: M Sears, McMaster University; Chile: V Aguirre, Hospital CRS El Pino; Ecuador: S Barba, AXXIS-Medical Centre, Sociedad Ecuatoriana de Alergologia, Inmunologia y Ciencias Afines [SEAICA]; India: J Shah, Jaslok Hospital & Research Centre; Indonesia: K Baratawidjaja, University of Indonesia; Japan: S Nishima, The National Minami-Fukuoka Chest Hospital; Malaysia: J de Bruyne, University of Malaya; Samoa: N Tuuau-Potoi, Ministry of Health, Samoa; SAR China: CK Lai, The Chinese University of Hong Kong; Singapore: BW Lee, National University of Singapore; Sudan: A El Sony, Epidemiological Laboratory (Epi-Lab) for Public Health, Research and Development; United Kingdom, Isle of Man: R Anderson, Population Health Research Institute, St George’s, University of London.
