# Supplementary material for: Are Environmental Factors for Atopic Eczema in ISAAC Phase Three due to Reverse Causation?
Source: J Invest Dermatol. 2019 May;139(5):1023–36. doi: 10.1016/j.jid.2018.08.035 (PMC6478380; doi:10.1016/j.jid.2018.08.035)
Supplement: Supplementary Methods and Supplementary Tables S1-S5 [file mmc1.pdf]

Supplementary Material

Supplementary methods

Centre and school eligibility criteria

Only centres that met ISAAC methodology standards were included in the analysis. Excluded centres were those with fewer than 1,000 participants or response rates below 60% for the 6-7 year-old age group or below 70% for the 13-14 year-olds. Centres were also excluded if they did not return the Centre Report (Ellwood et al., 2005). Schools with fewer than 10 participants for a given age group were excluded from that analysis.

Environmental risk factors

Analyses in this paper included only the key environmental variables previously each singly associated with AE in ISAAC at an individual level. For the 6-7 year olds, risk factors were paracetamol use in the first year of life and in the past 12 months (Beasley et al., 2008), antibiotic use in the first year of life (Foliaki et al., 2009), breast feeding (Bjorksten et al., 2011), cat and dog in the home in the first year of life (Brunekreef et al., 2012a), regular contact with farm animals in the first year of life (Brunekreef et al., 2012b), regular maternal contact with farm animals while pregnant (Brunekreef et al., 2012b), heavy truck traffic (Brunekreef et al., 2009), fast food consumption (Ellwood et al., 2013), parental smoking (Mitchell et al., 2012), cooking on an open fire (Wong et al., 2013), birthweight (Mitchell et al., 2014) and number of siblings (Strachan et al., 2015). For the 13-14 year olds, risk factors were heavy truck traffic (Brunekreef et al., 2009), fast food consumption (Ellwood et al., 2013), parental smoking (Mitchell et al., 2012), paracetamol use in the past 12 months (Beasley et al., 2011), open fire cooking (Wong et al., 2013), and number of siblings (Strachan et al., 2015).

Most of these items had simple “yes/no” answers. The exceptions have been dichotomised: paracetamol use in the last 12 months (at least once per month vs. less than once per month), heavy truck traffic (frequently or almost the whole day vs. seldom or never), fast food consumption (once per week or more vs. less than once per week), low birthweight (less than 2.5 kg vs. at least 2.5 kg), and number of siblings (2 or more vs. 1 or fewer). Full definitions of the environmental risk factors are in Table S2 of the Supplementary Material.

Derivation of affluence

Gross National Income (GNI) as of 2002 (obtained from the World Bank website (The World Bank, 2016a) where available and filled in by the Central Intelligence Agency (CIA) World Factbook (Central Intelligence Agency, 2002)) and a classification of affluent countries (GNI over US$9,205) and non-affluent countries (GNI US$9,205 or lower) taken from the 2001 World Bank definition of high-income countries versus low- to middle-income countries (The World Bank, 2016b).

Supplementary tables

Table S1: Summary statistics in subjects with data for atopic eczema symptoms, sex, level of maternal education and the one exposure of interest (the "maximum sample").

Age group	Variable	Individual-level		School-level		
		n	Prevalence (%)	n	Median prevalence (%)	Prevalence IQR (%)
6-7 years	Atopic eczema in the last 12 months	204,771	7.4	2,851	6.7	(2.9, 11.6)
	Farm animals (in utero)	181,600	10.0	2,630	7.8	(3.4, 15.6)
	Low birthweight	169,993	8.5	2,549	6.3	(3.2, 10.6)
	Paracetamol (1st year)	182,134	65.2	2,583	70.0	(56.3, 82.6)
	Antibiotics (1st year)	180,799	54.0	2,663	55.9	(46.2, 64.6)
	Breastfed ever	192,559	80.0	2,701	84.6	(73.6, 92.9)
	Cat (1st year)	189,922	12.0	2,701	10.5	(4.8, 20.0)
	Dog (1st year)	174,772	20.6	2,469	22.2	(12.3, 32.8)
	Farm animals (1st year)	181,744	11.6	2,634	9.8	(4.6, 17.8)
	2 or more siblings	203,603	38.1	2,851	37.5	(22.2, 54.4)
	Heavy Truck traffic (current)	184,503	38.6	2,729	38.2	(27.8, 49.1)
	Fast food (current)	181,864	41.1	2,798	33.3	(18.5, 51.1)
	Paternal tobacco (current)	196,353	31.3	2,748	33.3	(19.3, 46.5)
	Maternal tobacco (current)	199,522	14.1	2,781	14.0	(3.0, 30.1)
	Paracetamol (current)	191,900	19.8	2,734	16.7	(7.9, 31.3)
	Open fire cooking (current)	185,718	3.0	2,724	0.0	(0.0, 2.2)
13-14 years	Atopic eczema in the last 12 months	341,577	6.8	2,477	5.5	(2.7, 10.2)
	2 or more siblings	334,708	55.2	2,402	62.1	(38.9, 81.0)
	Heavy Truck traffic (current)	309,621	39.6	2,348	39.4	(30.3, 51.0)
	Fast food (current)	313,066	55.2	2,388	55.2	(40.7, 69.5)
	Paternal tobacco (current)	301,502	37.6	2,259	36.7	(23.9, 48.2)
	Maternal tobacco (current)	329,659	18.1	2,433	18.3	(4.0, 33.3)
	Paracetamol (current)	314,005	28.8	2,404	31.0	(19.5, 43.5)
	Open fire cooking (current)	303,363	7.3	2,321	1.1	(0.0, 4.7)

Table S2. Minimally adjusted[A] effects of individual- and school-level exposures on atopic eczema symptoms in the last 12 months in subjects with data for atopic eczema symptoms, sex, level of maternal education and the one exposure of interest (the “maximum sample”). Mixed logistic regression models with random intercepts at the school, centre and country levels.

Age group	Exposure	Individual-level exposure		School-level exposure	
		n	OR (95% CI)	n	OR (95% CI)
6-7 years	Farm animals (in utero)	181,600	1.37 (1.29, 1.45)	181,600	1.85 (1.39, 2.45)
	Low birthweight	169,993	0.93 (0.86, 1.00)	169,993	1.65 (1.07, 2.55)
	Paracetamol (1st year)	182,134	1.55 (1.48, 1.62)	182,134	1.12 (0.87, 1.44)
	Antibiotics (1st year)	180,799	1.60 (1.53, 1.66)	180,799	1.29 (1.00, 1.66)
	Breastfed ever	192,559	1.09 (1.04, 1.14)	192,559	0.94 (0.69, 1.26)
	Cat (1st year)	189,922	1.27 (1.21, 1.34)	189,922	1.79 (1.33, 2.41)
	Dog (1st year)	174,772	1.16 (1.11, 1.21)	174,772	1.48 (1.14, 1.92)
	Farm animals (1st year)	181,744	1.41 (1.34, 1.49)	181,744	2.06 (1.56, 2.72)
	2 or more siblings	203,603	0.98 (0.94, 1.01)	203,603	1.44 (1.20, 1.73)
	Heavy Truck traffic (current)	184,503	1.19 (1.14, 1.23)	184,503	1.15 (0.94, 1.39)
	Fast food (current)	181,864	1.03 (0.99, 1.07)	181,864	0.84 (0.69, 1.02)
	Paternal tobacco (current)	196,353	1.11 (1.07, 1.16)	196,353	1.27 (1.01, 1.60)
	Maternal tobacco (current)	199,522	1.17 (1.11, 1.22)	199,522	1.61 (1.24, 2.09)
	Paracetamol (current)	191,900	1.60 (1.53, 1.67)	191,900	1.69 (1.28, 2.23)
	Open fire cooking (current)	185,718	1.14 (1.02, 1.29)	185,718	2.98 (1.83, 4.85)
Age group	Exposure	Individual-level exposure		School-level exposure	
		n	OR (95% CI)	n	OR (95% CI)
13-14 years	2 or more siblings	334,708	1.06 (1.03, 1.10)	334,708	1.25 (0.99, 1.57)
	Heavy Truck traffic (current)	309,621	1.31 (1.27, 1.35)	309,621	1.53 (1.20, 1.96)
	Fast food (current)	313,066	1.09 (1.06, 1.13)	313,066	1.85 (1.48, 2.32)
	Paternal tobacco (current)	301,502	1.22 (1.18, 1.26)	301,502	0.72 (0.54, 0.96)
	Maternal tobacco (current)	329,659	1.23 (1.19, 1.28)	329,659	0.91 (0.66, 1.24)
	Paracetamol (current)	314,005	1.57 (1.52, 1.62)	314,005	2.14 (1.59, 2.88)
	Open fire cooking (current)	303,363	1.43 (1.34, 1.53)	303,363	1.50 (1.09, 2.06)

[A]Adjusted for sex and mothers level of education.

Table S3. Fully adjusted[A] effects of individual-level exposures on atopic eczema symptoms in the last 12 months in subjects with data for atopic eczema symptoms, sex, maternal education and all exposures of interest (the "common sample"), stratified by country-level affluence. Mixed logistic regression models with random intercepts at the school, centre and country levels.

Age group	Exposure	Affluent Countries		Non-Affluent countries		Effect modification
		Number exposed (%)	OR (95% CI)	Number exposed (%)	OR (95% CI)	
6-7 years	Farm animals (in utero)	2,970 (6.8)	1.00 (0.84, 1.20)	6,365 (8.2)	1.19 (1.06, 1.35)	<0.001
	Low birthweight	2,508 (5.8)	0.92 (0.80, 1.06)	6,763 (8.7)	0.85 (0.76, 0.96)	0.51
	Paracetamol (1st year)	27,222 (62.8)	1.29 (1.17, 1.41)	52,751 (68.1)	1.30 (1.21, 1.40)	0.93
	Antibiotics (1st year)	22,736 (52.4)	1.44 (1.34, 1.55)	44,504 (57.5)	1.37 (1.28, 1.47)	0.33
	Breastfed ever	29,658 (68.4)	1.16 (1.07, 1.25)	67,532 (87.2)	1.05 (0.97, 1.15)	0.11
	Cat (1st year)	6,717 (15.5)	1.01 (0.92, 1.10)	6,508 (8.4)	1.21 (1.09, 1.33)	<0.001
	Dog (1st year)	8,102 (18.7)	0.98 (0.90, 1.06)	15,790 (20.4)	1.09 (1.02, 1.17)	<0.001
	Farm animals (1st year)	3,688 (8.5)	0.96 (0.81, 1.13)	7,662 (9.9)	1.29 (1.15, 1.44)	<0.001
	2 or more siblings	12,887 (29.7)	0.95 (0.89, 1.03)	29,066 (37.5)	0.93 (0.87, 1.00)	0.95
	Heavy Truck traffic (current)	14,353 (33.1)	1.07 (1.00, 1.15)	31,515 (40.7)	1.14 (1.07, 1.21)	0.14
	Fast food (current)	13,496 (31.1)	1.05 (0.97, 1.13)	34,343 (44.4)	0.96 (0.89, 1.02)	0.10
	Paternal tobacco (current)	16,991 (39.2)	1.05 (0.98, 1.14)	21,468 (27.7)	1.04 (0.97, 1.11)	0.40
	Maternal tobacco (current)	12,058 (27.8)	1.00 (0.92, 1.08)	7,620 (9.8)	1.15 (1.05, 1.26)	0.008
	Paracetamol (current)	5,011 (11.6)	1.64 (1.49, 1.79)	16,724 (21.6)	1.35 (1.25, 1.45)	0.003
	Open fire cooking (current)	255 (0.6)	1.09 (0.73, 1.63)	2,000 (2.6)	1.10 (0.92, 1.33)	0.60

Age group	Exposure	Affluent Countries		Non-Affluent Countries		Effect modification
		Number exposed (%)	OR (95% CI)	Number exposed (%)	OR (95% CI)	
13-14 years	2 or more siblings	18,086 (37.2)	1.06 (0.97, 1.16)	108,122 (58.6)	1.08 (1.03, 1.13)	0.51
	Heavy Truck traffic (current)	17,725 (36.5)	1.32 (1.21, 1.44)	74,568 (40.4)	1.31 (1.26, 1.37)	0.81
	Fast food (current)	24,780 (51.0)	1.06 (0.97, 1.15)	100,139 (54.3)	1.06 (1.01, 1.10)	0.79
	Paternal tobacco (current)	19,486 (40.1)	1.05 (0.96, 1.14)	70,043 (38.0)	1.17 (1.12, 1.23)	0.01
	Maternal tobacco (current)	14,713 (30.3)	1.09 (0.99, 1.20)	27,899 (15.1)	1.12 (1.06, 1.18)	0.31
	Paracetamol (current)	13,211 (27.2)	1.75 (1.60, 1.92)	49,682 (26.9)	1.53 (1.47, 1.60)	0.007
	Open fire cooking (current)	513 (1.1)	1.55 (1.10, 2.18)	11,565 (6.3)	1.44 (1.30, 1.60)	0.73

[A]Adjusted for sex, mother's level of education and all other variables in the table.

Table S4: Fully adjusted[A] effects of school-level exposures on prevalence on atopic eczema symptoms in the last 12 months in subjects with data for atopic eczema symptoms, sex, maternal education and all exposures of interest (the "common sample"), stratified by country-level affluence. Mixed logistic regression models with random intercepts at the school, centre and country levels.

Age group	Exposure	Affluent countries		Non-affluent countries		Effect modification p-value
		Median prevalence (%)	OR (95% CI)	Median prevalence (%)	OR (95% CI)	
6-7 years	Farm animals (in utero)	5.7	1.51 (0.56, 4.03)	7.0	0.77 (0.30, 1.97)	0.13
	Low birthweight	5.1	1.16 (0.49, 2.73)	6.3	1.87 (0.94, 3.70)	0.19
	Paracetamol (1st year)	76.0	1.29 (0.82, 2.05)	68.2	0.88 (0.58, 1.34)	0.28
	Antibiotics (1st year)	56.6	1.39 (0.91, 2.12)	58.3	1.23 (0.79, 1.90)	0.41
	Breastfed ever	74.1	1.15 (0.77, 1.71)	89.1	0.83 (0.45, 1.54)	0.35
	Cat (1st year)	10.0	1.02 (0.64, 1.64)	7.7	1.45 (0.68, 3.09)	0.03
	Dog (1st year)	18.8	0.79 (0.51, 1.24)	20.6	1.18 (0.72, 1.92)	0.03
	Farm animals (1st year)	7.8	0.57 (0.22, 1.47)	8.6	1.86 (0.81, 4.31)	0.02
	2 or more siblings	27.8	1.01 (0.72, 1.40)	36.4	1.08 (0.77, 1.51)	0.44
	Heavy Truck traffic (current)	33.3	0.83 (0.60, 1.14)	40.4	0.83 (0.60, 1.16)	0.77
	Fast food (current)	27.3	0.79 (0.54, 1.17)	35.7	1.11 (0.81, 1.52)	0.34
	Paternal tobacco (current)	41.5	1.02 (0.67, 1.55)	29.4	0.61 (0.38, 0.99)	0.57
	Maternal tobacco (current)	30.0	1.43 (0.96, 2.14)	7.7	1.88 (1.01, 3.51)	0.54
	Paracetamol (current)	11.7	2.05 (1.23, 3.42)	16.9	1.35 (0.83, 2.22)	0.38
	Open fire cooking (current)	0.0	2.75 (0.40, 18.79)	0.0	1.62 (0.80, 3.27)	0.97
Age group	Exposure	Affluent countries		Non-affluent countries		Effect modification p-value
		Median prevalence (%)	OR (95% CI)	Median prevalence (%)	OR (95% CI)	
13-14 years	2 or more siblings	34.8	1.26 (0.76, 2.10)	67.7	1.33 (0.97, 1.82)	0.16
	Heavy Truck traffic (current)	37.0	0.99 (0.53, 1.84)	40.0	1.47 (1.10, 1.97)	0.12
	Fast food (current)	50.0	1.40 (0.80, 2.43)	54.9	2.42 (1.84, 3.20)	0.03
	Paternal tobacco (current)	42.9	0.45 (0.21, 0.95)	34.0	0.73 (0.47, 1.12)	0.16
	Maternal tobacco (current)	35.3	1.46 (0.68, 3.12)	13.4	0.64 (0.39, 1.04)	0.48
	Paracetamol (current)	30.2	2.38 (1.26, 4.51)	29.4	2.62 (1.79, 3.85)	0.58
	Open fire cooking (current)	0.0	1.08 (0.04, 26.81)	0.7	2.26 (1.40, 3.66)	0.45

[A]Adjusted for sex, mother's level of education and all other variables in the table.

Table S5: Risk factor definitions

Risk factors for ages 6-7	Question (asked to parent)	Positive Response
Farm animals (in utero)	Has the child's mother had regular (at least once a week) contact with farm animals (e.g. cattle, pigs, goats, sheep or poultry) while being pregnant with this child?	Yes
Low birthweight	What was the weight of your child when he / she was born?	Less than 2.5kg
Paracetamol (1st year)	In the first 12 months of your child's life, did you usually give paracetamol for fever?	Yes
Antibiotics (1st year)	In the first 12 months of your child's life, did your child have any antibiotics?	Yes
Breastfed ever	Was your child breastfed?	Yes
Cat (1st year)	Did you have a cat in your home during the first year of your child's life?	Yes
Dog (1st year)	Did you have a dog in your home during the first year of your child's life?	Yes
Farm animals (1st year)	In your child's first year of life, did he / she have regular (at least once a week) contact with farm animals (e.g. cattle, pigs, goats, sheep or poultry)?	Yes
2 or more siblings	How many older and younger brothers and sisters does your child have?	Total of 2 or more
Heavy truck traffic (current)	How often do trucks pass through the street where you live, on weekdays?	Frequently or almost the whole day
Fast food (current)	In the past 12 months, how often, on average did your child eat fast food / burgers?	At least once a week
Paternal tobacco (current)	Does your child's father (or male guardian) smoke cigarettes?	Yes
Maternal tobacco (current)	Does your child's mother (or female guardian) smoke cigarettes?	Yes
Paracetamol (current)	In the past 12 months, how often, on average, have you given your child paracetamol?	At least once a month
Open fire cooking (current)	In your house, what fuels are usually used for cooking? Electricity, Gas, Open fires, Other	Any that include open fires

Risk factors for ages 13-14	Question (asked to child)	Positive Response
2 or more siblings	How many older and younger brothers and sisters do you have?	Total of 2 or more
Heavy truck traffic (current)	How often do trucks pass through the street where you live, on weekdays?	Frequently or almost the whole day
Fast food (current)	In the past 12 months, how often, on average did you eat fast food / burgers?	At least once a week
Paternal tobacco (current)	Does your father (or male guardian) smoke cigarettes?	Yes
Maternal tobacco (current)	Does your mother (or female guardian) smoke cigarettes?	Yes
Paracetamol (current)	In the past 12 months, how often, on average, have you taken paracetamol?	At least once a month
Open fire cooking (current)	In your house, what fuels are usually used for cooking? Electricity, Gas, Open fires, Other	Any that include open fires
